# Enhanced analysis of gating latency in 0.35T MR‐linac through innovative time synchronization of a motion phantom and plastic scintillation detector

**DOI:** 10.1002/acm2.70116

**Published:** 2025-05-13

**Authors:** Mateb Al Khalifa, Tianjun Ma, Haya Aljuaid, Siyong Kim, William Y. Song

**Affiliations:** ^1^ Department of Radiation Oncology Virginia Commonwealth University Richmond Virginia USA

**Keywords:** beam on/off latency, dose per pulse, MRgRT, MR‐linac, plastic scintillation detector, QA

## Abstract

**Purpose:**

This study aims to evaluate how different gantry angles, breathing rates (BPM), cine image speeds, and tracking algorithms affect beam on/off latency and the subsequent impact on target dose for a 0.35T MR‐Linac with a 6 MV FFF beam. The investigation incorporates an image‐based MRI4D modus QA motion phantom (MQA) and a measurement‐based plastic scintillation detector (PSD).

**Methods:**

The MQA's target was customized with an insertion for a 1 mm PSD from BluePhysics. Both the PSD and the MQA were simultaneously synchronized to the Linac to capture latency signals. A plan was created in the ViewRay TPS to deliver dose to the target at three gantry angles (0°, 120°, and 240°). Each gantry angle was evaluated at three breathing rates (10, 12, and 15 BPM). The study also examined two imaging speeds (4 and 8 FPS) and four tracking algorithms.

**Results:**

Across all configurations at 4 FPS, the overall mean beam‐on latency was 0.339 ± 0.06 s from the PSD and 0.318 ± 0.06 s from the MQA, whereas at 8 FPS it was 0.630 ± 0.07 s (PSD) and 0.609 ± 0.07 s (MQA). Conversely, the overall mean beam‐off latency at 4 FPS was 0.153 ± 0.03 s (PSD) and 0.124 ± 0.03 s (MQA), while at 8 FPS it was 0.121 ± 0.06 s (PSD) and 0.205 ± 0.04 s (MQA). The overall mean difference between gating and non‐gating doses was an increase of 12.050 ± 9.2 cGy at 4 FPS and 14.044 ± 7.4 cGy at 8 FPS.

**Conclusion:**

This comprehensive study underscores the significant influence of gantry angle, breathing rate, cine imaging speed, and tracking algorithms on latency and dose delivery accuracy in a 0.35T MR‐Linac.

## 1 INTRODUCTION

The primary goal of radiotherapy is to deliver the prescribed dose to the tumor to improve tumor control probability while minimizing the dose to surrounding normal tissue to reduce normal tissue complication probability. Tumor motion during radiotherapy presents challenges that must be appropriately managed to achieve treatment goals. Inaccurate dose delivery due to tumor motion can potentially lead to tumor control failure and increased normal tissue complications. Different organs exhibit varying degrees of motion during treatment. Complex respiratory motion involves physiological processes that can significantly affect tumor position in both inter‐ and intra‐fractionation.^1^, ^2^, ^3^ The target can move by a substantial range and may not be uniform across consecutive breathing cycles.^4^, ^5^ However, target motion exceeding 5 mm is generally recommended to be managed during radiotherapy treatment.^6^, ^7^

Various motion‐management techniques are available, including the application of an internal target volume (ITV) margin as recommended in International Commission on Radiation Units and Measurements (ICRU) Report 62.6 to account for tumor motion, shape, and positional changes during both inter‐ and intra‐fractional variations.^8^ Other techniques include breath holding,^9^, ^10^, ^11^ abdominal compression,^12^ 4D‐CT scan encompassing,^13^, ^14^, ^15^ real‐time Multileaf Collimator (MLC) tracking,^16^, ^17^, ^18^ and real‐time gated imaging.^19^, ^20^, ^21^ Additionally, external surrogates and implanted fiducial markers can be used for motion management, although they present challenges. For instance, external surrogates can be inaccurate because the surrogate's motion may become unsynchronized with the actual tumor position over time.^22^, ^23^ Meanwhile, using internal fiducial markers is not suitable for all anatomical sites and carries the potential for complications; moreover, capturing target motion and its shifted position in real time may be difficult.^24^, ^25^, ^26^

Magnetic resonance–guided radiotherapy (MRgRT) is an effective option for image‐guided radiotherapy (IGRT) because it integrates magnetic resonance imaging (MRI) with a linear accelerator (MR‐linac), allowing continuous imaging of patient anatomy during treatment. Compared to X‐ray–based imaging, MRI gating provides superior soft tissue contrast without additional imaging dose.^27^, ^28^, ^29^, ^30^ These advantages of MRgRT have demonstrated high local control rates with minimal toxicity for certain disease sites.^31^, ^32^, ^33^

The efficiency of gating and real‐time target tracking depends on the Linac control system and the latency of the gated imaging process.^1^, ^34^ Gating latency is the time delay between initiation or termination of the beam‐control signal and the corresponding change in beam status.^35^ Many factors affect latency, including image acquisition speed, processing time, target‐tracking algorithm performance, and radiation beam control delays. High gating latency can introduce systematic errors in delivered dose relative to the planned dose.

Several studies have evaluated and estimated gating latency using two broad approaches: (1) employing dosimetric devices and (2) measuring beam‐control signals directly. In the first approach, dosimetric devices such as film or plastic scintillation detectors can be used to measure the dose during gating and estimate latency from the resulting dose distributions.^36^, ^37^, ^38^, ^39^ In the second approach, gating latency is measured by comparing the timing of the Linac beam‐control signal with the motion‐management control signal, thus capturing the time delay between the gating trigger and the corresponding change in beam status; no dosimetric device is required in this method.^40^, ^41^

In this study, we evaluate the effect of beam‐on and beam‐off latency on target dose in a 6 MV FFF 0.35 T MR‐Linac (ViewRay Inc., Oakwood Village, Ohio). Our approach combines an image‐based MRI4D Modus QA phantom (MQA) (ModusQA, Ontario, Canada) with a dose‐based, high‐temporal‐and‐spatial‐resolution Plastic Scintillation Detector (PSD) from BluePhysics (Lutz, Florida, USA). Notably, we have, for the first time, successfully integrated signals from: (1) the 0.35 T MR‐Linac radiation control system, (2) the MQA system, and (3) the PSD controllers onto a single timeframe to comprehensively capture latency in milliseconds and delivered dose in cGy. We analyze beam‐on and beam‐off latency across two cine image frame rates, three breathing rates, three different gantry angles, and four tracking algorithms. Using the PSD's per‐pulse measurements, we also evaluate latency not only through imaging but via the recorded dose pulses themselves, enabling a thorough assessment of the tracking algorithms. This comprehensive study characterizes the effects of gating latency on the dose delivered to the target and includes a unique first‐hand account of dose measurements under various gating conditions.

## 2 MATERIALS AND METHODS

The dose from gated treatment in a 0.35 T MR‐Linac is generally affected by the beam‐trigger signal processing time, image acquisition speed, and target‐tracking algorithms. In this study, a complete record of timestamps for the radiation beam‐control signals, the MQA controller signals, and the PSD signals was documented and analyzed.

### 2.1 0.35 T MRI‐guided radiotherapy system

A 0.35 T MRgRT system (ViewRay Inc., Oakwood Village, Ohio) was used to assess dose‐gating latency measurements. The system is composed of a 0.35 T split‐doughnut superconducting MRI capable of real‐time MR imaging, with space in the split design to house a 6 MV flattening‐filter‐free (FFF) LINAC system.^42^ The MRI sequence protocol used for clinical imaging and treatment planning is a 3D steady‐state precession (TRUFI) pulse sequence. TRUFI is acquired in the axial orientation for localizing treatment targets, while a 2D TRUFI cine MRI in the sagittal orientation is used for real‐time gated imaging during treatment.^41^

### 2.2 Cine MRI specification

The cine MRI can track tumor motion during treatment at time resolutions of 0.25 s (4 FPS) and 0.125 s (8 FPS). This 2D low‐resolution real‐time MR imaging uses deformable image registration (DIR) for target and organ contour deformation.^43^, ^44^ Compressed sensing is implemented for image reconstruction from incoherently undersampled k‐space.^45^ While compressed sensing rapidly increases the frame rate, it may introduce artifacts such as blurring and global ringing, thus reducing image quality.^46^ Consequently, increasing the cine frame rate from 4 FPS to 8 FPS comes at the cost of reduced signal‐to‐noise ratio (SNR).

### 2.3 Quasar 4D moving phantom

The MRI4D QUASAR is an MR‐safe, programmable motion phantom with an efficient piezoelectric motor assembly that can be imaged with high quality and minimal artifacts. The phantom's oval body (20 cm L × 30 cm W × 20 cm H) contains two insert cavities (centered and offset) designed to hold acrylic inserts that can be filled with distilled water. An interchangeable acrylic stem insert (25 cm L × 7.4 cm internal diameter) includes a central cuboid target customized for targeting and gating imaging. This cuboid target (3 × 4 × 5 cm^3^) has a centrally located hole designed to accommodate the PSD. The MQA application is used to control the insert's motion, thereby moving the cuboid target for both dosimetric and imaging measurements.

The oval phantom body is mounted on a treatment couch indexing cradle designed to accommodate the array coils. It is connected to the drive unit rod and the insert. An MR‐safe cable (quadruple‐shielded DB‐15 male‐to‐female) connects the phantom drive unit on the treatment couch to an MR‐safe DB‐15 port‐saver connector through the waveguide, ensuring a secure connection between the two motor cables in the radiofrequency (RF) cabinet. A brass sponge is placed around the MR‐safe DB‐15 port‐saver connector inside the waveguide to prevent RF interference. The electronic control box is powered via an AC power cord (110–220 V, 50–60 Hz) and provides a DC power supply. It also includes a CAT5e Ethernet port for connection to a laptop in the control area.

### 2.4 BluePhysics plastic scintillation detector

The BluePhysics plastic scintillation detector (Lutz, Florida, USA), model 10, uses a BCF‐10 scintillation fiber (Saint‐Gobain, Hiram, Ohio, USA). The scintillator itself measures 1 mm × 1 mm (length × diameter) and has a volume of 0.785 mm^3^. The optical fiber is made of polymethyl methacrylate with a fluoridated polymer cladding, measuring 0.25 mm in diameter; its total length is 10 m from detector tip to electrometer reader. This PSD employs a subtraction method to remove Cerenkov radiation from the scintillation signal, as described by Ferrer et al.^47^ It can sample at 1.4 kHz (700 µs). With a maximum dose rate corresponding to a pulse every 5 ms, it can acquire 12 samples between consecutive pulses. This sampling strategy allows measurement of every single pulse from the 0.35 T MR‐Linac with a temporal accuracy of ±700 µs from pulse arrival to the detector. The PSD also has a dedicated port for receiving the 0.35 T MR‐Linac beam‐control signal (beam on/off).

The PSD calibration is required to isolate the plastic scintillation signal from the optical fiber's Cerenkov component (the Cerenkov correction). First, the PSD is irradiated in a PTW 1D water tank under reference conditions (9.96 cm × 9.96 cm field, source‐to‐surface distance of 80 cm, depth = *d*_max_ = 1.4 cm) to deliver 1 MU = 1 cGy. The detector is irradiated with 100 MU to calibrate for 100 cGy. The calibration factor is obtained by measuring the charge that is proportional to dose under these reference conditions. The charge‐proportional‐to‐dose is calculated as

(1) $$\begin{array}{ccc} & & Charge\;Proportional\;to\;Dose\;\left[nC\right]\\ & & =Sensor\;Charge-\left[Cerenkov\;Charge\;x\;ACR\right]\; \end{array}$$

where ACR is the adjacent channel ratio, found by

(2) $$ACR=\frac{R_{chPSD,\;9.96x9.96}\;\times \;OF-\;R_{chPSD,\;3.32x3.32}}{R_{chCR,\;9.96x9.96\;}\times \;OF-\;R_{chCR,\;3.32x3.32}}$$

$R_{chPSD,\;9.96x9.96}$ and $R_{chPSD,\;3.32x3.32}$ are the sensor charge readings at field sizes of 9.96 cm × 9.96 cm and 3.32 cm × 3.32 cm, respectively. Similarly, $R_{chCR,\;9.96x9.96\;}$ and $R_{chCR,\;3.32x3.32}$ are the Cerenkov fiber charge readings at those field sizes. OF is the output factor for the 3.32 cm × 3.32 cm field relative to the 9.96 cm × 9.96 cm reference field, taken from the ViewRay MRIdian TPS. The calibration factor is then:

(3) $$Calibration\;Factor=\frac{Dose\;\left[cGy\right]}{Charge\;Proportional\;to\;Dose\left[nC\right]}$$

Each measured total charge under given delivery conditions is multiplied by this calibration factor to obtain the dose.

### 2.5 Treatment planning

The MQA oval body was scanned using a 3D steady‐state precession (TRUFI) pulse sequence. The imaging parameters were TR = 3.37 ms, TE = 1.45 ms, flip angle = 60°, imaging matrix = 300 × 334, field of view (read × phase) = 500 × 449.1 mm^2^, slice thickness = 1.5 mm, and rBW = 535 Hz/pixel. The scan was performed in a head‐first supine orientation, yielding 288 MR images. Contouring was done for the phantom body and the cubic target used for gating. The isocenter was placed at the center of the cubic target. Three fixed conformal beams were planned for three gantry angles: 0° (381.7 MU), 120° (420.2 MU), and 240° (414.4 MU). No CT scan was performed; dose calculations were carried out on the MRI scan by forcing the oval body and insert to have an electron density of 1 (water‐equivalent). The dose calculation used 2,400,000 histories and a dose grid resolution of 0.3 cm.

### 2.6 Synchronization among the three systems

The MQA can record beam‐control signals from the 0.35 T MR‐linac. A gating window is set in the MQA software through specified trigger points, which are then compared to the beam on/off signal from the MR‐Linac to calculate gating latency. Simultaneously, the MQA software can capture the timestamps at which the 0.35 T MR‐linac is switched on or off, along with the target motion timestamps from the MQA drive unit. Latency is thus defined as the time difference between the target position and the Linac beam‐delivery timestamp. The MQA software calculates latency for all target motion cycles (entering and exiting the gating window) and the associated beam on/off signals from the 0.35 T MR‐linac.

The PSD and its electrometer measure the dose per pulse. By noting the first and last pulses detected by the PSD once the target is in range, it is possible to compute the time intervals between those pulses and the Linac's on/off signals. As a result, on/off latency can be recorded among the three systems: the PSD, the MQA, and the 0.35 T MR‐linac.

**FIGURE 1:**
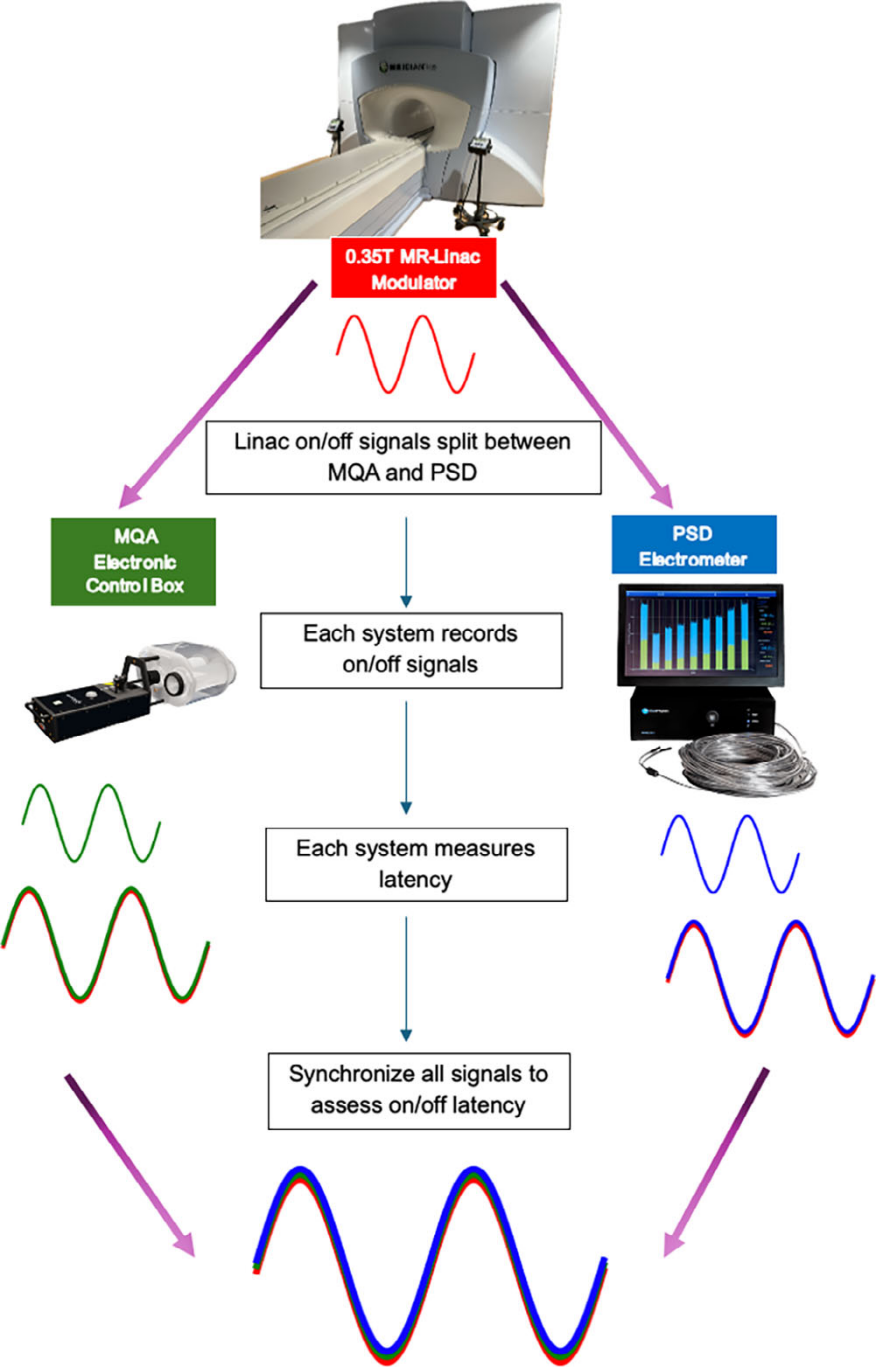
A diagram showing how the linac on/off signal line is split into two branches: one going to the PSD electrometer and the other to the MQA electronic control box. It also illustrates how each system ultimately latches the signal for synchronization.

To accurately synchronize all three systems, it is necessary to use the same timeframe for on/off signal measurements. To achieve this, the beam on/off signal from the 0.35 T MR‐linac was split so that when the Linac sends a beam on/off signal, it is transmitted simultaneously to both the MQA control box and the PSD electrometer, as shown in Figure 1. The PSD electrometer was specifically modified in this study to receive the Linac beam‐control signal.

### 2.7 Treatment setup and delivery

An MRI scan was performed to verify the phantom's position. The TRUFI sequence was used (FOV: 50 × 45 × 43 cm^3^, total scan time = 172 s). Registration was carried out between the MRI data from the TPS and the setup images. An isocenter shift was applied to precisely position the phantom so that the MQA cubic gating target lay within the treatment field.

To enable real‐time gating on the sagittal plane, a clearly visible cubic target representation was chosen to define the motion boundary. This boundary extended the target by 3 cm in all directions except longitudinally (−*Y*/+*Y*), which was extended by 0.8 cm. The gating field of view was 35 cm × 35 cm, with a resolution of 0.243 cm × 0.70 cm. The region of interest (ROI) threshold; defined as the fraction (by volume or pixel count) of the contoured target allowed to exceed the gating boundary before triggering beam hold; was set to 0%, ensuring that any part of the target leaving the boundary would instantly pause the beam. The optimal frame was identified, and update key frame was selected.

In the MQA software, the intersection of the target contour and the tracking boundary was fine‐tuned to maintain a minimal target out % (<2%) and a consistent “Target out of bounds” indication (∼5 s). This process establishes the triggers for the target boundaries in the longitudinal direction (−*Y*/+*Y*). The procedure was repeated for both 4 FPS and 8 FPS.

Two sets of deliveries were performed for each gantry angle (0°, 120°, and 240°) at three breathing rates (10, 12, and 15 BPM): (1) a non‐gated delivery and (2) a gated delivery. These were repeated for two imaging speeds (4 FPS and 8 FPS) and four tracking algorithms (default, small mobile targets [SMT], large deforming targets [LDT], and large deforming targets [CMDT]). Data were recorded and analyzed in both the MQA and the PSD systems.

## 3 RESULTS

### 3.1 Synchronization among the three systems

**FIGURE 2:**
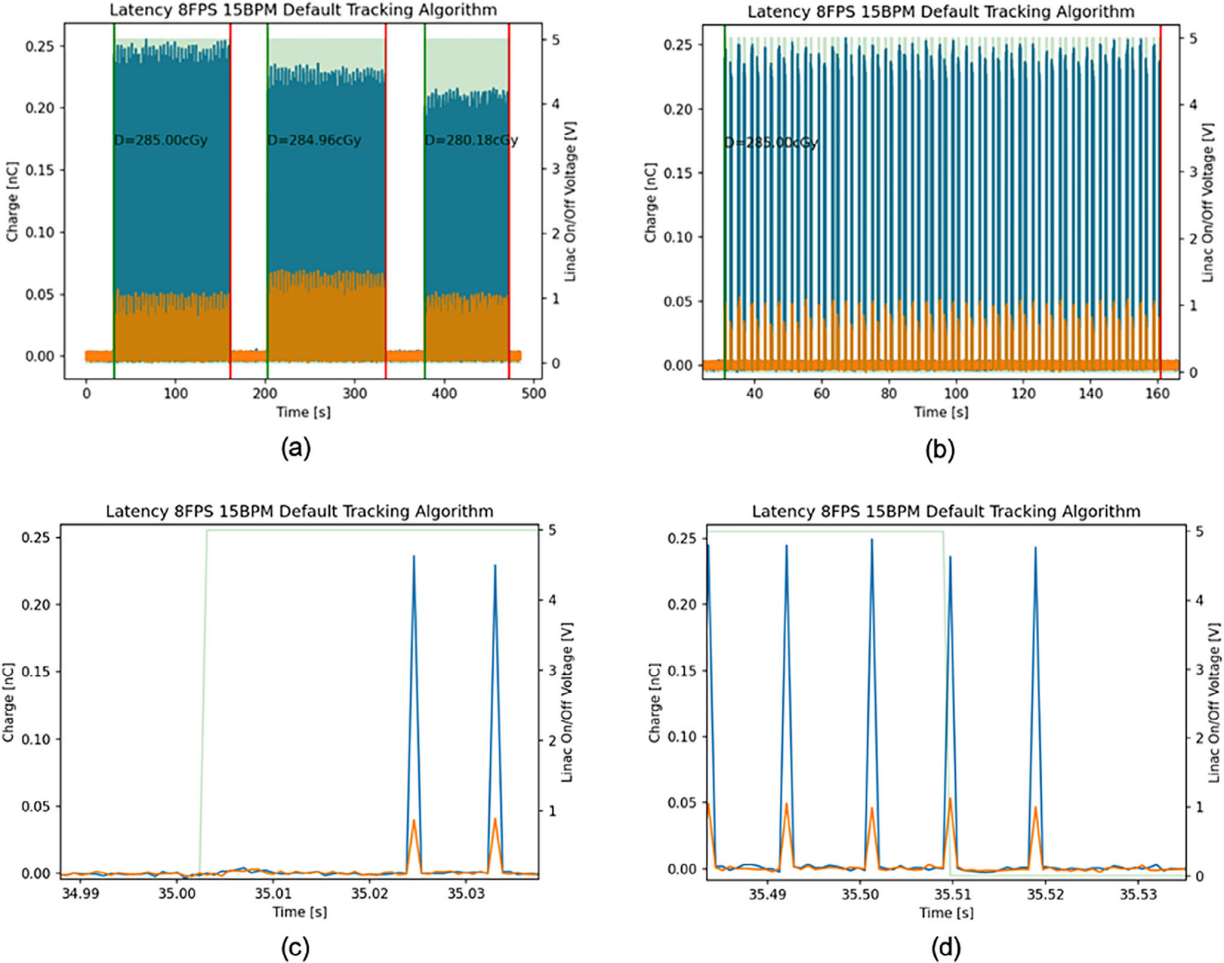
Exhibiting the measurement performance by the PSD. (a) A representative example of one complete treatment delivery, showing the synchronization of the beam signal map from the 0.35 T MR‐Linac (green background), representing the Linac activation window, and the PSD charge pulses (blue spikes). The graph also shows Cerenkov radiation (orange spikes) from the scintillator detector, arising from the interaction between the radiation and the PSD during gating. The horizontal axis represents the time scale from the start to the end of the beam delivery. The left vertical axis represents the charge from the PSD, and the right vertical axis represents the Linac voltage for beam on/off signals. The graph contains three windows corresponding to three delivered gantry angles (0°, 120°, and 240°, from left to right). The green and red bold lines between each field represent the start and end of that delivery field, respectively. The dose from each gantry angle, also shown in cGy, can be derived from subtracting the Cerenkov charge. (b) A closer look at the 0° gantry angle delivery window, showing the number of delivered cycles and the superimposed PSD pulses with the 0.35 T MR‐Linac beam on/off signals. (c) A closer look at the first pulse, showing the beam‐on latency, defined as the distance between the Linac activation window's beam‐on line and the first delivered pulse. (d) Shows the last pulse when the beam was off. The beam‐off latency here is the distance between the Linac activation window's beam‐off line and the last delivered pulse. This example corresponds to an 8 FPS, 15 BPM delivery using the Default tracking algorithm.

Figure 2 displays a sample signal map from the PSD and the 0.35 T MR‐Linac for a complete treatment delivery example. Figure 2a shows a plot of synchronization between pulses, recorded as charge and Cerenkov radiation from the PSD, and the 0.35 T MR‐linac beam signals. Three windows represent three gantry angles delivered at 0°, 120°, and 240°. Figure 2b exhibits a closer look at one gantry angle (0° in this case), showing the number of cycles delivered for each gantry angle. Figure 2c shows the pulses from the PSD that were delivered in each cycle within the linac activation window. It illustrates the beam‐on latency determined by the PSD, which is defined as the distance between the first pulse and the beam‐on line of the linac activation window. Figure 2d shows the beam‐off latency measured by the PSD, which is defined as the distance between the beam‐off line of the Linac activation window and the last pulse from the PSD.

**FIGURE 3:**
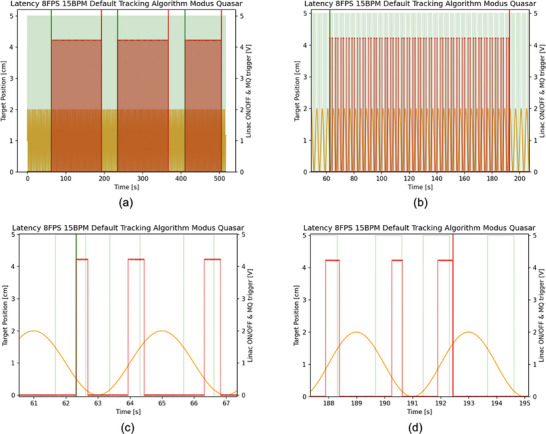
Exhibiting the measurement performance by the MQA. (a) A representative example of one complete treatment delivery, showing synchronization of the beam signal map from the 0.35 T MR‐Linac (light green background) with the MQA (light red windows). The graph also depicts the target's motion (orange waves). The horizontal axis represents the time scale in seconds, from the start to the end of beam delivery. The left vertical axis shows the target position in centimeters, while the right vertical axis shows the Linac on/off signal and the trigger in volts. The graph contains three windows (0°, 120°, and 240° gantry angles, from left to right). The green and red bold lines between each field represent the start and end of each field. No dose is shown for each gantry angle in this figure. (b) A closer look at the 0° gantry angle window, showing the number of the MQA target‐motion cycles recorded and the superimposed cycling of the MQA with the 0.35 T MR‐Linac beam on/off signals. (c) A closer look at the first MQA target cycle, defining beam‐on latency as the distance between the beam‐on line of the Linac activation window (light green rectangle) and the first MQA target‐position cycle (light red rectangle). (d) The last MQA target‐position cycle with the beam off, where the off latency is the distance between the beam‐off line of the Linac activation window (light green rectangle) and the final MQA target‐position cycle (light red rectangle). This example corresponds to an 8 FPS, 15 BPM delivery using the Default tracking algorithm.

Figure 3 demonstrates the latency measurement recorded by the MQA. Figure 3a shows an example of one complete treatment delivery, displaying the synchronization of the 0.35 T MR‐Linac beam signal map with the MQA and the target's motion. The graph contains three windows, corresponding to the three gantry angles 0°, 120°, and 240°, from left to right, with a light green background indicating the Linac activation window and the MQA trigger combined. It is worth noting that the MQA system does not display dose for each gantry angle. Figure 3b provides a closer look at a gantry angle delivery window (0° in this case), showing the number of the MQA target‐position cycles recorded. It depicts the superimposed cycling from the MQA with the 0.35 T MR‐Linac beam on/off signals. Figure 3c focuses on the first MQA target‐position cycle to define beam‐on latency as the distance between the start of the Linac activation window (when the beam turned on) and the first recorded MQA target‐position cycle. Figure 3d shows the last MQA target‐position cycle with the beam off; here, off latency is defined as the distance between the end of the Linac activation window (when the beam turned off) and the last MQA target‐position cycle recorded.

**FIGURE 4:**
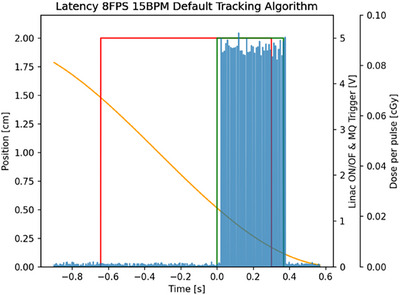
A representative example of a single cycle, during which the object moves forward, illustrating the overlap among the target motion from the MQA (green rectangular window), the PSD pulses (blue pulses), and the 0.35 T MR‐Linac activation window (red rectangular window). The horizontal axis shows time in seconds, from the start of beam delivery through one cycle. Negative time values refer to the period before delivery starts, and the zero value indicates when the MQA drive unit records the gating target entering the field (and the motion signal is reset to zero). Time = 0 represents the timestamp at which the three systems are synchronized for each cycle. The left vertical axis shows the gating target's position (in cm), controlled by the MQA drive unit. The first right vertical axis shows the Linac voltage for the beam on/off signals and the MQA trigger (in V). The second right vertical axis shows dose pulses from the PSD (in cGy).

**FIGURE 5:**
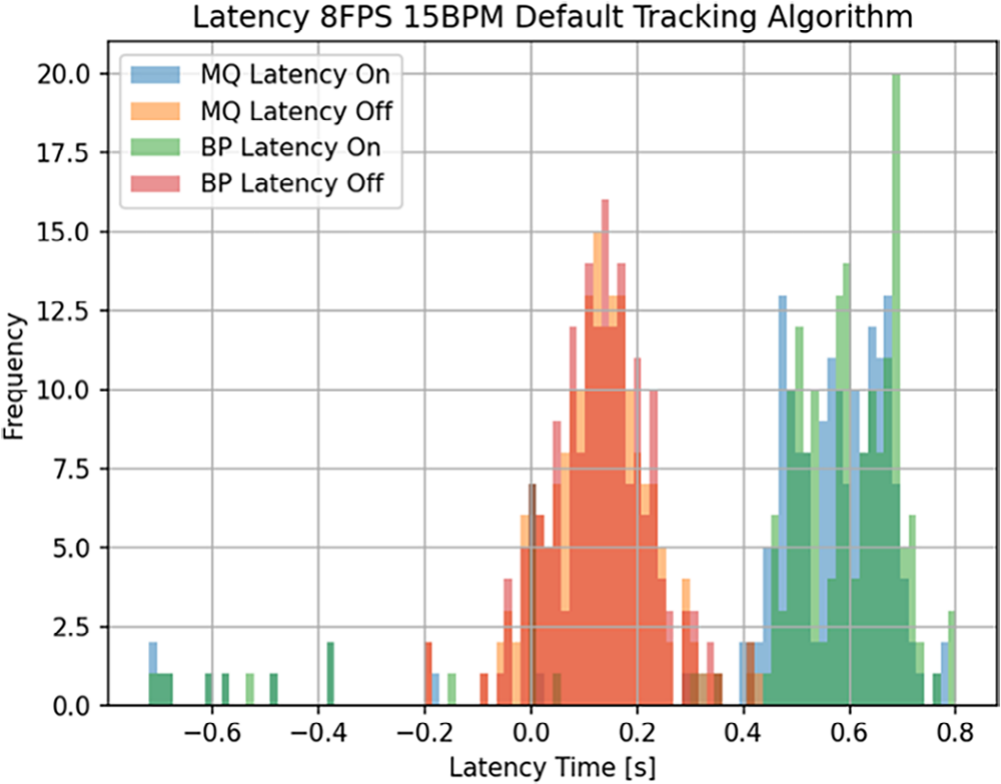
A representative histogram for the sample treatment delivery that illustrates the latency measurements across the MQA and the PSD. The horizontal axis represents latency in seconds, while the vertical axis represents the frequency of these measurements. Light blue and green bars correspond to the frequency of beam‐on signals for the MQA and the PSD, respectively, and the light orange and red bars show the frequency of beam‐off signals for the MQA and the PSD, respectively. “MQ” and “BP” in the legend stand for the MRI4D Modus QA motion phantom and the BluePhysics PSD, respectively. This sample histogram represents an 8 FPS, 15 BPM delivery using the Default tracking algorithm.

Figure 4 illustrates the overall overlap among the three systems, showing the 0.35 T MR‐linac activation window alongside both the MQA and the PSD activation windows. The plots indicate beam‐on latency for both systems as the distance from the initial line of the 0.35 T MR‐linac activation window, whereas beam‐off latency is measured from the end of that window. The plot represents an example of a single cycle in which the object moves forward. Figure 5 presents a sample histogram for latency measurements recorded by both the MQA and the PSD. It provides a comparative view of latency associated with the 0.35 T MR‐linac beam on/off signals for a representative delivery (8 FPS, 15 BPM, Default tracking). The histogram is typical of all configurations, showing that beam‐on latency is generally higher than beam‐off latency.

### 3.2 Gating latency measurement

**FIGURE 6:**
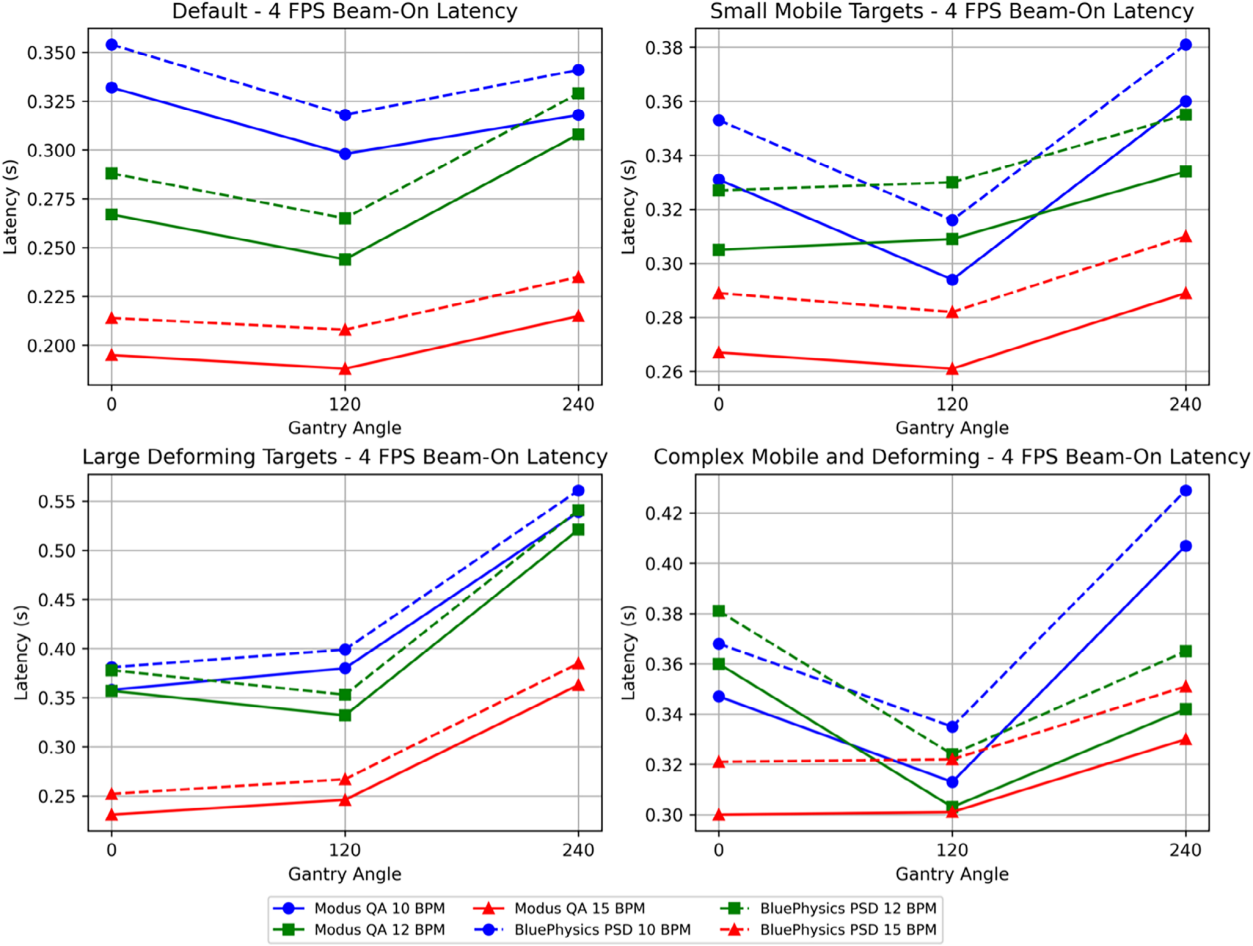
Plots showing the beam‐on latency measured by the MQA and the PSD at 4 FPS with various tracking algorithms, gantry angles, and BPM values. The horizontal axis represents the gantry angle, and the vertical axis represents the on latency in seconds.

**FIGURE 7:**
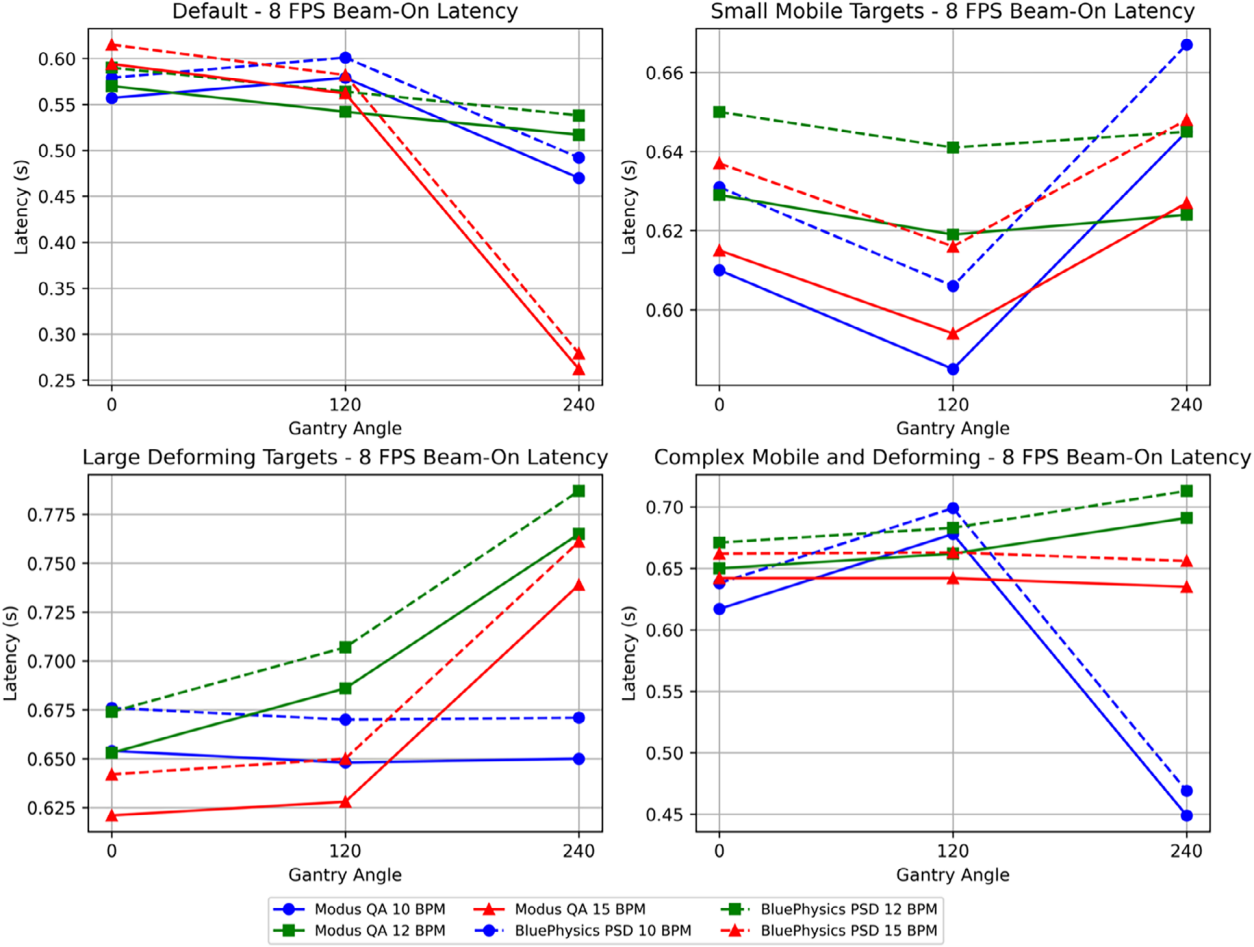
Plots showing the beam‐on latency measured by the MQA and the PSD at 8 FPS with various tracking algorithms, gantry angles, and BPM values. The horizontal axis represents the gantry angle, and the vertical axis represents the on latency in seconds.

Beam on/off latency for the 0.35 T MR‐Linac was measured by both the MQA and the PSD for four tracking algorithms and two cine image speeds (4 FPS and 8 FPS) across three gantry angles (0°, 120°, 240°) and three breathing rates (10, 12, 15 BPM). Table 1 presents the beam‐on latency using the PSD measurements. The mean beam‐on latency across all configurations was 0.339 ± 0.06 s at 4 FPS and 0.630 ± 0.07 s at 8 FPS. Table 2 shows the beam‐on latency measured using the MQA, which generally indicates slightly lower values than the PSD, with a mean beam‐on latency of 0.318 ± 0.06 s at 4 FPS and 0.609 ± 0.07 s at 8 FPS. The difference between the PSD and the MQA beam‐on latency ranged from a minimum of 0.017 s to a maximum of 0.023 s, with an average difference of 0.021 ± 0.0009 s over all configurations. Figures 6 and 7 illustrate beam‐on latency measured by both methods at 4 FPS and 8 FPS, respectively.

**TABLE 1:** Beam‐on latency (in seconds) from the PSD measurements for four tracking algorithms, three motion rates, three gantry angles, and two cine imaging speeds.

		4 FPS				8 FPS			
Tracking algorithms	Gantry angle	10 BPM	12 BPM	15 BPM	Mean (s)	10 BPM	12 BPM	15 BPM	Mean (s)
Default	0	0.354	0.288	0.214	0.285 ± 0.07	0.579	0.590	0.615	0.594 ± 0.02
	120	0.318	0.265	0.208	0.263 ± 0.06	0.601	0.564	0.582	0.582 ± 0.02
	240	0.341	0.329	0.235	0.302 ± 0.06	0.492	0.538	0.279	0.437 ± 0.14
Small mobile targets	0	0.353	0.327	0.289	0.323 ± 0.03	0.631	0.650	0.637	0.639 ± 0.01
	120	0.316	0.330	0.282	0.309 ± 0.02	0.606	0.641	0.616	0.621 ± 0.02
	240	0.381	0.355	0.310	0.349 ± 0.04	0.667	0.645	0.648	0.653 ± 0.01
Large deforming targets	0	0.381	0.378	0.252	0.337 ± 0.07	0.676	0.674	0.642	0.664 ± 0.02
	120	0.399	0.353	0.267	0.340 ± 0.07	0.670	0.707	0.650	0.675 ± 0.03
	240	0.561	0.541	0.385	0.496 ± 0.10	0.671	0.787	0.761	0.739 ± 0.06
Complex mobile and deforming	0	0.368	0.381	0.321	0.357 ± 0.03	0.638	0.671	0.662	0.657 ± 0.02
	120	0.335	0.324	0.322	0.327 ± 0.01	0.699	0.683	0.663	0.682 ± 0.02
	240	0.429	0.365	0.351	0.382 ± 0.04	0.469	0.713	0.656	0.613 ± 0.13
Mean (s)		0.378 ± 0.07	0.353 ± 0.07	0.286 ± 0.05	0.339 ± 0.06	0.616 ± 0.07	0.655 ± 0.07	0.618 ± 0.11	0.630 ± 0.07

**TABLE 2:** Beam‐on latency (in seconds) from the MQA measurements for four tracking algorithms, three motion rates, three gantry angles, and two cine imaging speeds.

Tracking algorithms	Gantry angle	4 FPS				8 FPS			
		10 BPM	12 BPM	15 BPM	Mean (s)	10 BPM	12 BPM	15 BPM	Mean (s)
Default	0	0.332	0.267	0.195	0.264 ± 0.07	0.557	0.570	0.594	0.573 ± 0.02
	120	0.298	0.244	0.188	0.243 ± 0.06	0.579	0.542	0.562	0.561 ± 0.02
	240	0.318	0.308	0.215	0.280 ± 0.06	0.470	0.517	0.262	0.416 ± 0.14
Small mobile targets	0	0.331	0.305	0.267	0.301 ± 0.03	0.610	0.629	0.615	0.618 ± 0.01
	120	0.294	0.309	0.261	0.288 ± 0.02	0.585	0.619	0.594	0.599 ± 0.02
	240	0.360	0.334	0.289	0.328 ± 0.04	0.645	0.624	0.627	0.632 ± 0.01
Large deforming targets	0	0.358	0.357	0.231	0.315 ± 0.07	0.654	0.653	0.621	0.643 ± 0.02
	120	0.380	0.332	0.246	0.319 ± 0.07	0.648	0.686	0.628	0.654 ± 0.03
	240	0.539	0.521	0.363	0.475 ± 0.10	0.650	0.765	0.739	0.718 ± 0.06
Complex mobile and deforming	0	0.347	0.360	0.300	0.336 ± 0.03	0.617	0.650	0.642	0.636 ± 0.02
	120	0.313	0.303	0.301	0.306 ± 0.01	0.678	0.662	0.642	0.660 ± 0.02
	240	0.407	0.342	0.330	0.360 ± 0.04	0.449	0.691	0.635	0.592 ± 0.13
Mean (s)		0.356 ± 0.07	0.332 ± 0.07	0.265 ± 0.05	0.318 ± 0.06	0.595 ± 0.07	0.634 ± 0.07	0.597 ± 0.11	0.609 ± 0.07

**FIGURE 8:**
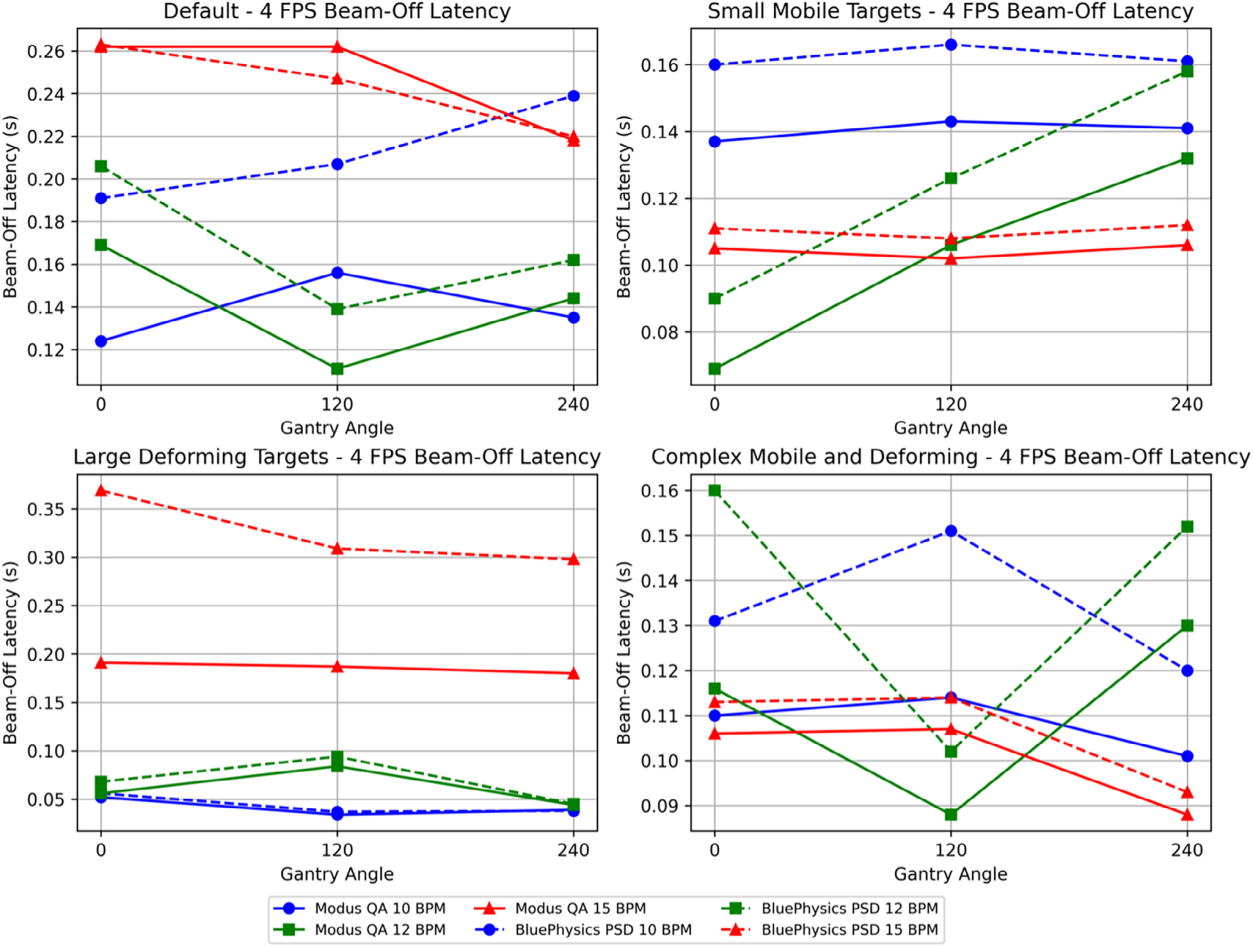
Plots showing the beam‐off latency measured by the MQA and the PSD at 4 FPS with various tracking algorithms, gantry angles, and BPM values. The horizontal axis represents the gantry angle, and the vertical axis represents the off latency in seconds.

**FIGURE 9:**
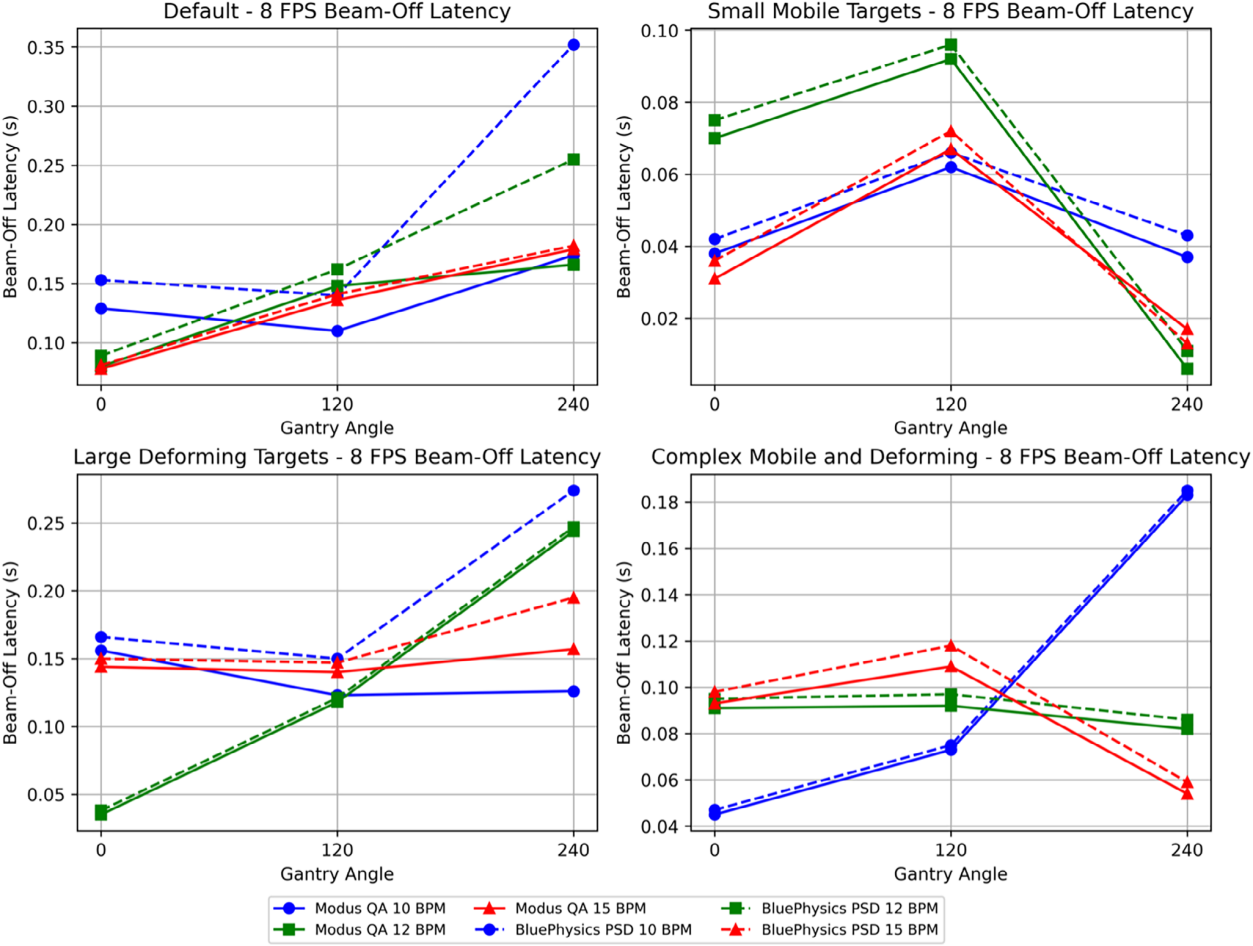
Plots showing the beam‐off latency measured by the MQA and the PSD at 8 FPS with various tracking algorithms, gantry angles, and BPM values. The horizontal axis represents the gantry angle, and the vertical axis represents the off latency in seconds.

The mean beam‐off latency measured by the PSD across all configurations was 0.153 ± 0.03 s at 4 FPS and 0.121 ± 0.06 s at 8 FPS (Table 3). Table 4 shows the MQA‐measured beam‐off latency, with means of 0.124 ± 0.03 s at 4 FPS and 0.205 ± 0.04 s at 8 FPS. The difference between the MQA and the PSD beam‐off latency ranged from 0.016 s to 0.178 s. The average difference across all configurations was 0.024 ± 0.04 s. Figures 8 and 9 depict beam‐off latency for all configurations at 4 FPS and 8 FPS, respectively.

**TABLE 3:** Beam‐off latency (in seconds) from the PSD measurements for four tracking algorithms, three motion rates, three gantry angles, and two cine imaging speeds.

Tracking algorithms	Gantry angle	4 FPS				8 FPS			
		10 BPM	12 BPM	15 BPM	Mean (s)	10 BPM	12 BPM	15 BPM	Mean (s)
Default	0	0.191	0.206	0.263	0.220 ± 0.04	0.153	0.089	0.081	0.108 ± 0.04
	120	0.207	0.139	0.247	0.197 ± 0.05	0.140	0.162	0.141	0.147 ± 0.01
	240	0.239	0.162	0.220	0.207 ± 0.04	0.352	0.255	0.182	0.263 ± 0.09
Small mobile targets	0	0.160	0.090	0.111	0.120 ± 0.04	0.042	0.075	0.036	0.051 ± 0.02
	120	0.166	0.126	0.108	0.133 ± 0.03	0.066	0.096	0.072	0.078 ± 0.02
	240	0.161	0.158	0.112	0.144 ± 0.03	0.043	0.011	0.013	0.022 ± 0.02
Large deforming targets	0	0.056	0.068	0.369	0.164 ± 0.18	0.166	0.038	0.150	0.118 ± 0.07
	120	0.037	0.094	0.309	0.147 ± 0.14	0.150	0.121	0.147	0.139 ± 0.02
	240	0.038	0.045	0.298	0.127 ± 0.15	0.274	0.247	0.195	0.239 ± 0.04
Complex mobile and deforming	0	0.131	0.160	0.113	0.135 ± 0.02	0.047	0.095	0.098	0.080 ± 0.03
	120	0.151	0.102	0.114	0.122 ± 0.03	0.075	0.097	0.118	0.096 ± 0.02
	240	0.120	0.152	0.093	0.121 ± 0.03	0.185	0.086	0.059	0.110 ± 0.07
Mean (s)		0.138 ± 0.07	0.125 ± 0.05	0.196 ± 0.10	0.153 ± 0.03	0.141 ± 0.10	0.114 ± 0.07	0.108 ± 0.06	0.121 ± 0.06

**TABLE 4:** Beam‐off latency (in seconds) from the MQA measurements for four tracking algorithms, three motion rates, three gantry angles, and two cine imaging speeds.

Tracking algorithms	Gantry angle	4 FPS				8 FPS			
		10 BPM	12 BPM	15 BPM	Mean (s)	10 BPM	12 BPM	15 BPM	Mean (s)
Default	0	0.124	0.169	0.262	0.185 ± 0.07	0.129	0.080	0.078	0.095 ± 0.03
	120	0.156	0.111	0.262	0.177 ± 0.08	0.110	0.148	0.136	0.132 ± 0.02
	240	0.135	0.144	0.218	0.166 ± 0.05	0.174	0.166	0.179	0.173 ± 0.01
Small mobile targets	0	0.137	0.069	0.105	0.103 ± 0.03	0.038	0.070	0.031	0.046 ± 0.02
	120	0.143	0.106	0.102	0.117 ± 0.02	0.062	0.092	0.067	0.074 ± 0.02
	240	0.141	0.132	0.106	0.126 ± 0.02	0.037	0.006	0.017	0.020 ± 0.02
Large deforming targets	0	0.052	0.056	0.191	0.100 ± 0.08	0.156	0.035	0.144	0.111 ± 0.07
	120	0.034	0.084	0.187	0.102 ± 0.08	0.123	0.118	0.140	0.127 ± 0.01
	240	0.039	0.044	0.180	0.088 ± 0.08	0.126	0.244	0.157	0.176 ± 0.06
Complex mobile and deforming	0	0.110	0.116	0.106	0.111 ± 0.01	0.045	0.091	0.093	0.077 ± 0.03
	120	0.114	0.088	0.107	0.103 ± 0.01	0.073	0.092	0.109	0.091 ± 0.02
	240	0.101	0.130	0.088	0.106 ± 0.02	0.183	0.082	0.054	0.106 ± 0.07
Mean (s)		0.107 ± 0.04	0.104 ± 0.04	0.159 ± 0.07	0.124 ± 0.03	0.105 ± 0.05	0.102 ± 0.06	0.100 ± 0.05	0.205 ± 0.04

### 3.3 Dose gating measurement

The dose delivered to the target, as measured by the PSD placed at the center of the MQA's cubic gating target, without gating was 282.1, 280.0, and 272.7 cGy for gantry angles 0°, 120°, and 240°, respectively, yielding a total of 834.6 cGy across all three angles. Table 5 shows the gated‐dose results for different tracking algorithms at 4 FPS and 8 FPS, for the three gantry angles (0°, 120°, 240°) and three breathing rates (10, 12, 15 BPM). The mean dose from all configurations was 282.2 ± 4.5 cGy at 4 FPS and 282.9 ± 4.8 cGy at 8 FPS. Table 6 presents the total gated dose derived from these values. The mean total dose from all configurations was 846.7 ± 9.2 cGy at 4 FPS and 848.7 ± 7.4 cGy at 8 FPS.

**TABLE 5:** Gating dose (in cGy) from the PSD measurements for four tracking algorithms, three motion rates, three gantry angles, and two cine imaging speeds.

Tracking algorithms	Gantry angle	4 FPS				8 FPS			
		10 BPM	12 BPM	15 BPM	Mean (cGy)	10 BPM	12 BPM	15 BPM	Mean (cGy)
Default	0	280.250	281.000	280.940	280.7 ± 0.42	290.970	289.310	285.000	288.4 ± 3.08
	120	278.060	279.330	278.800	278.7 ± 0.64	288.200	284.370	284.960	285.8 ± 2.06
	240	272.460	272.820	273.480	272.9 ± 0.52	282.150	279.800	280.180	280.7 ± 1.26
Small mobile targets	0	286.740	287.500	287.470	287.2 ± 0.43	289.440	285.710	283.240	286.1 ± 3.12
	120	286.340	285.850	285.580	285.9 ± 0.39	287.760	281.690	283.580	284.3 ± 3.11
	240	279.810	278.980	279.990	279.6 ± 0.54	281.490	272.060	272.130	275.2 ± 5.42
Large deforming targets	0	283.000	286.650	287.480	285.7 ± 2.38	288.720	287.590	285.390	287.2 ± 1.69
	120	281.870	286.150	284.630	284.2 ± 2.17	286.470	285.970	284.990	285.8 ± 0.75
	240	277.900	278.400	278.240	278.2 ± 0.26	278.670	276.390	279.030	278.0 ± 1.43
Complex mobile and deforming	0	288.260	286.580	288.140	287.7 ± 0.94	285.520	285.020	282.770	284.4 ± 1.46
	120	285.880	284.620	286.410	285.6 ± 0.92	283.260	284.590	280.920	282.9 ± 1.86
	240	278.610	279.380	282.420	280.1 ± 2.01	275.920	274.480	276.210	275.5 ± 0.93
Mean (cGy)		281.6 ± 4.64	282.3 ± 4.59	282.8 ± 4.59	282.2 ± 4.01	284.9 ± 4.63	282.2 ± 5.45	281.5 ± 4.09	282.9 ± 4.05

**TABLE 6:** Total gating dose (in cGy) from the PSD measurements for four tracking algorithms, three motion rates, three gantry angles, and two cine imaging speeds.

Tracking algorithms	Gantry angle	4 FPS				8 FPS			
		10 BPM	12 BPM	15 BPM	Mean (cGy)	10 BPM	12 BPM	15 BPM	Mean (cGy)
Default	0	830.770	833.150	833.220	832.4 ± 1.39	861.320	853.480	850.140	855.0 ± 5.74
	120								
	240								
Small mobile targets	0	852.890	852.330	853.040	852.8 ± 0.37	858.690	839.460	838.950	845.7 ± 11.3
	120								
	240								
Large deforming targets	0	842.770	851.200	850.350	848.1 ± 4.64	853.860	849.950	849.410	851.1 ± 2.43
	120								
	240								
Complex mobile and deforming	0	852.750	850.580	856.970	853.4 ± 3.25	844.700	844.090	839.900	842.9 ± 2.61
	120								
	240								
Mean (cGy)		844.8 ± 10.5	846.8 ± 9.1	848.4 ± 10.5	846.7 ± 7.0	854.6 ± 7.3	846.7 ± 6.2	844.6 ± 6.0	848.7 ± 4.9

## 4 DISCUSSION

In this study, we investigated gating dose and gating latency in a 0.35 T MR‐Linac using both the PSD and the MQA, synchronizing the data recorded from both systems. We included all tracking algorithms available in the 0.35 T MR‐linac (Default, SMT, LDT, and CMDT). Each of these algorithms targets a specific type of motion. The Default algorithm can be applied to most targets (except the stomach). The SMT algorithm is used for small targets, such as the pancreas or metastatic liver lesions with large motion and slight deformation. The LDT algorithm applies to larger targets, such as the prostate or bladder, where there is slight motion but extensive deformation. The CMDT algorithm is intended for targets experiencing large deformation and translational motion, for example in gynecological sites.^40^ Because the efficiency of each tracking algorithm affects target detection during routine cine imaging, we investigated all four algorithms for their impact on gating latency and dose.^40^

We also examined how target motion speed affects gating latency and dose. Target motion speed can influence image quality (e.g., motion blurring), which can affect the accuracy of tracking algorithms. Three motion speeds (10, 12, and 15 BPM) were implemented via the MQA, with the PSD inserted to measure dose. These three rates were selected to represent different possible organ motion speeds. Since real organ motion may not be perfectly cyclic or constant, we also reported data averaged across these three speeds to show how variable motion affects gating latency and dose.

Three gantry angles (0°, 120°, and 240°) were employed to assess the role of beam angle on gating latency and dose. The 120° and 240° angles can be influenced by couch passage, whereas the 0° angle does not. Additionally, geometric distortion at different gantry angles may affect image quality, and thus tracking accuracy and gating latency. Further, two real‐time cine imaging speeds (4 and 8 FPS) were tested to evaluate whether view‐sharing at 8 FPS impacts gating dose and latency.

Traditionally, researchers have investigated latency effects on dose distributions using two commercially available MRI‐compatible moving phantoms: the MRI4D QUASAR phantom,^40^, ^48^, ^49^, ^50^, ^51^, ^52^, ^53^, ^54^ and the CIRS thorax phantom (CIRS, Inc., Norfolk, VA, USA).^36^, ^55^, ^56^ Some authors have developed in‐house targets compatible with these motion phantoms,^39^, ^56^ while others have fabricated custom phantoms.^57^, ^58^, ^59^ There is also interest in using dose‐based, high‐temporal‐and‐spatial‐resolution plastic scintillation detectors to study gating.^38^ A clinical investigation by Kim et al. described using the MQA to measure latency in a 0.35 T MR‐Linac, finding an average beam‐off latency of 189 ± 25 ms at 15 BPM, 128 ± 23 ms at 12 BPM, and 161 ± 23 ms at 10 BPM. Their work introduced a clinical implementation of the MRI4D QUASAR phantom, using a single software platform to control the moving target and log the gating signal without relying on an oscilloscope. By contrast, an older method required an oscilloscope and physical sensor, as in the study by Green et al., who reported an average beam‐off latency of 394 ms (ranging from 246 to 527 ms) for 15 cine on a 0.35 T MR–60Co system.^40^, ^41^ Other investigators have used film measurements to estimate latency analytically, without directly accessing the Linac's electrical signals. Nakayama et al. found a beam‐off latency of 0.51 ± 0.17 s and a beam‐on latency of 0.35 ± 0.05 s, illustrating an alternate approach.^39^

We took a novel approach by splitting the on/off trigger signals from the 0.35 T MR‐Linac so they could be sent simultaneously to both the MQA and the PSD (see Figure 1). Because the on/off signals came from a single source (the 0.35 T MR‐Linac), the MQA and the PSD each received identical timing information. The MQA software holds the gating‐target position information, establishing a gating window that turns the beam on or off according to the target's motion. The PSD was integrated into the MQA's gating target to measure dose per pulse. Whenever the target moved in or out of the gating window (as shown in Figure 4), the PSD recorded the first pulse and the last pulse. We computed the interval between when the beam‐on signal was triggered by the Linac and when the first pulse arrived at the PSD (Figure 2c), defining the beam‐on latency. Similarly, beam‐off latency was defined as the time from when the Linac beam‐off signal was issued until the last detected pulse (Figure 2d).

Initially, we synchronized the MQA and the PSD signals from the first gating‐target cycle and extended that synchronization to all cycles. However, we discovered a clock drift between the MQA and the PSD that affected subsequent cycles. To address this, we resynchronized for each cycle (Figure 4). Because the Linac on/off signals serve as a common link between the MQA and the PSD, once resynchronized for each cycle, beam‐on latency became the interval from the MQA gating trigger to the PSD's first detected pulse. Similarly, beam‐off latency was the interval from the MQA gating trigger to the PSD's last detected pulse. Although the Linac digital signals are 5 V for “on” and 0 V for “off,” whereas the PSD's digital logic uses 3 V of 5 V logic, the system performed without issue. The PSD's photodetector requires a specific optimal operating voltage, which the PSD software regulates automatically.

Our results showed that beam‐on latency was generally higher at 8 FPS than at 4 FPS for all configurations, in both the MQA and the PSD data, indicating that a higher frame rate did not reduce beam‐on latency. The PSD tended to measure slightly higher beam‐on latency than the MQA, likely due to different measurement mechanisms. Across all configurations, the beam‐on latency difference between the PSD and the MQA ranged from 0.017 s to 0.023 s (mean 0.021 ± 0.0009 s). The Default algorithm yielded the lowest beam‐on latency at both 4 and 8 FPS among the four algorithms. At 15 BPM, the beam‐on latency was lowest for 4 FPS across all algorithms, whereas at 8 FPS no consistent pattern emerged. Gantry angle did not show a clear effect on beam‐on latency. Clinically, beam‐on latency is less critical because it occurs when the target is moving into the gating window.

Beam‐off latency is more consequential because it extends the time the target might be outside the gating window. We found that beam‐off latency improved at 8 FPS compared to 4 FPS in most configurations, though a few showed the opposite. Overall, the PSD measured slightly higher beam‐off latency than the MQA, with differences ranging from 0.016 s to 0.178 s (mean 0.024 ± 0.04 s). Gantry angle did not show a consistent trend for beam‐off latency, though it varied among algorithms.

We also investigated gating dose. Overall, gating impacted the dose received by the target, reflected in differences between gated and non‐gated deliveries, for individual fields and total treatments (Tables 5 and 6). In some deliveries, gated doses surpassed non‐gated doses, indicating the effect of latency on delivered dose. In others, the non‐gated dose was slightly higher.

This study has several limitations. First, synchronization among the PSD, the MQA, and the 0.35T MR‐Linac was accomplished by separately coding for each cycle. A more efficient method might use a single clock (one computer controlling both the PSD and the MQA). Second, the cubic shape of the gating target is simplified compared to real clinical targets with more complex geometries. Third, the motion waveform used herein may not replicate the full complexity of clinical organ motion. Different tracking algorithms may excel with certain shapes or speeds. Fourth, this study used static‐field beam delivery rather than IMRT. Finally, we did not validate the TPS calculation by comparing the PSD results with TPS outputs; we only examined gated versus non‐gated dose.

As gating becomes more established in radiotherapy, further improvements could enhance image‐guided radiotherapy evaluation. Developing a 3D object with 3D dosimetric mapping for gating would allow more realistic assessments of both beam on/off latency and 3D dose distributions. Because advanced target motion patterns, shapes, and speeds that mirror actual clinical scenarios are needed for fully representative gating studies, a 3D system could incorporate boundary definitions tailored to different shapes, speeds, frame rates, and tracking algorithms. Ultimately, IMRT gating could be comprehensively evaluated for both dose distribution and on/off latency.

## 5 CONCLUSION

A novel approach was used to synchronize the beam on/off signals from the 0.35 T MR‐Linac between the MQA and the PSD. By leveraging the per‐pulse nature of the PSD and the MQA's capabilities as a gating‐testing phantom, we assessed both gating dose and beam on/off gating latency. These assessments were performed at three gantry angles, three target speeds, two cine imaging speeds, and for all tracking algorithms provided by the 0.35 T MR‐Linac. Our study introduces the concept of simultaneously directing the 0.35 T MR‐Linac beam on/off signal to different systems to evaluate latency not only with a target‐motion phantom but also with an independent dosimeter. Future work could involve measuring latency and gating dose in a full 3D target dose distribution for IMRT beam delivery, rather than limiting assessments to point dose or 2D distributions.

## AUTHOR CONTRIBUTIONS

Mateb Al Khalifa: Conceptualization, Acquisition, Interpretation of data, Methodology, Validation, Drafting the work, Revising the draft, Final approval of the version. Tianjun Ma: Conceptualization, Interpretation of data, Methodology, Validation, Revising the draft, Final approval of the version. Haya Aljuaid: Conceptualization, Interpretation of data, Methodology, Validation, Revising the draft, Final approval of the version. Siyong Kim: Conceptualization, Interpretation of data, Methodology, Validation, Revising the draft, Final approval of the version. William Y. Song: Conceptualization, Interpretation of data, Methodology, Validation, Revising the draft, Final approval of the version.

## CONFLICT OF INTERESTS TATEMENT

The authors declare no conflicts of interest.
